# Prolonged fasting followed by refeeding modifies proteome profile and parvalbumin expression in the fast-twitch muscle of pacu (*Piaractus mesopotamicus*)

**DOI:** 10.1371/journal.pone.0225864

**Published:** 2019-12-19

**Authors:** Rafaela Nunes da Silva-Gomes, Maria Laura Gabriel Kuniyoshi, Bruno Oliveira da Silva Duran, Bruna Tereza Thomazini Zanella, Paula Paccielli Freire, Tassiana Gutierrez de Paula, Bruno Evaristo de Almeida Fantinatti, Rondinelle Artur Simões Salomão, Robson Francisco Carvalho, Lucilene Delazari Santos, Maeli Dal-Pai-Silva

**Affiliations:** 1 Department of Morphology, Institute of Bioscience of Botucatu, São Paulo State University (UNESP), Botucatu, São Paulo, Brazil; 2 University of Western São Paulo (UNOESTE), Presidente Prudente, São Paulo, Brazil; 3 Center for the Studies of Venoms and Venomous Animals (CEVAP)/ Graduate Program in Tropical Diseases (FMB), São Paulo State University (UNESP), Botucatu, São Paulo, Brazil; University of Maryland Center for Environmental Science, UNITED STATES

## Abstract

Here, we analyzed the fast-twitch muscle of juvenile *Piaractus mesopotamicus* (pacu) submitted to prolonged fasting (30d) and refeeding (6h, 24h, 48h and 30d). We measured the relative rate of weight and length increase (RRI_length_ and RRI_weight_), performed shotgun proteomic analysis and did Western blotting for PVALB after 30d of fasting and 30d of refeeding. We assessed the gene expression of *igf-1*, *mafbx* and *pvalb* after 30d of fasting and after 6h, 24h, 48h and 30d of refeeding. We performed a bioinformatic analysis to predict miRNAs that possibly control parvalbumin expression. After fasting, RRI_length_, RRI_weight_ and *igf-1* expression decreased, while the *mafbx* expression increased, which suggest that prolonged fasting caused muscle atrophy. After 6h and 24h of refeeding, *mafbx* was not changed and *igf-1* was downregulated, while after 48h of refeeding *mafbx* was downregulated and *igf-1* was not changed. After 30d of refeeding, RRI_length_ and RRI_weight_ were increased and *igf-1* and *mafbx* expression were not changed. Proteomic analysis identified 99 proteins after 30d of fasting and 71 proteins after 30d of refeeding, of which 23 and 17, respectively, were differentially expressed. Most of these differentially expressed proteins were related to cytoskeleton, muscle contraction, and metabolism. Among these, parvalbumin (PVALB) was selected for further validation. The analysis showed that *pvalb* mRNA was downregulated after 6h and 24h of refeeding, but was not changed after 30d of fasting or 48h and 30d of refeeding. The Western blotting confirmed that PVALB protein was downregulated after 30d of fasting and 30d of refeeding. The downregulation of the protein and the unchanged expression of the mRNA after 30d of fasting and 30d of refeeding suggest a post-transcriptional regulation of PVALB. Our miRNA analysis predicted 444 unique miRNAs that may target *pvalb*. In conclusion, muscle atrophy and partial compensatory growth caused by prolonged fasting followed by refeeding affected the muscle proteome and PVALB expression.

## Introduction

Skeletal muscle comprises up to 60% of fish body weight and plays a crucial role in functions such as movement and support [1,2]. The growth and atrophy of skeletal muscle are finely controlled by anabolic and catabolic pathways. It is well established that the ubiquitin ligases *mafbx* and *murf1* are linked to muscle catabolism [3,4], while the insulin growth factor 1 (*igf-1*) activates the anabolic pathway, resulting in muscle growth. However, many aspects of these pathways remain to be unveiled, including those involved in muscle homeostasis [5,6] and muscle plasticity in response to intrinsic and extrinsic factors [7,8].

Fish are an attractive model to study skeletal muscle, on account of its importance for human nutrition as a protein source [9]. Another significant advantage is that unlikely mammals, their fast-twitch and slow-twitch fibers are separated in distinct compartments, which facilitates the analysis of a single muscle fiber type. The slow-twitch fibers usually are concentrated along the lateral line, while the fast-twitch muscle corresponds to a higher volume and presents a more central location [10].

One major extrinsic factor that influences muscle plasticity is food availability [8,11]. Experiments of fasting followed by refeeding have been used as a model of muscle atrophy and growth in several fish studies [12–16]. In general, fasting leads to activation of muscle catabolism [17] and inhibition of anabolism [18,19], resulting in muscle atrophy and body weight loss. In contrast, subsequent refeeding may trigger accelerated growth that partially, fully or overcompensate the growth inhibition during the fasting [14]. With exception of a previous gel-based proteomics study of our research group [16], there are no literature regarding the proteomics of fish muscle submitted to fasting and refeeding.

An important protein related to muscle function and homeostasis is the parvalbumin (PVALB). This calmodulin-like protein is the main allergen in seafood, and thus is a central subject in meat quality studies [20,21]. The PVALB also plays an important role in fast-twitch muscle relaxation and in the maintenance of calcium homeostasis [22–25]: since this protein binds to cytosolic Ca^2+^ and translocates it to the endoplasmic reticulum, it protects cells from cytosolic Ca^2+^ overload and facilitates muscle relaxation by diminishing Ca^2+^ binding to the tropomyosin-troponin complex. However, few studies have already analyzed parvalbumin expression in anabolic and catabolic conditions, especially in the muscle of non-mammalian vertebrates, and there is no research about parvalbumin in the muscle of fasted and refed fish.

In studies about muscle, proteomics is an exciting approach to provide an overview of all the proteins with changed abundance in response to a treatment and to environmental factors [26]. In particular, our research group is focused on global, broad-scale, discovery proteomics [27]. We believe that this approach permits the discovery of novel factors previously unknown to be involved in the situation of interest, what would not be possible with targeted proteomics, PCR and Western blot, which requires a previous selection of the target. In a previous publication, we used the two-dimensional electrophoresis to analyze the muscle of fish submitted to fasting and refeeding, since it is a more classic and mature approach that is particularly useful to study intact proteins and isoforms [16]. The two-dimensional electrophoresis can be complemented and enriched by shotgun proteomics, which is a newer and faster proteomic methodology [28,29]. Thus, a shotgun proteomic analysis can corroborate and broaden the reach of our previous research [16].

In this work, we departed from a more holistic and exploratory objective of identifying the proteome profile of the fast-twitch muscle of pacu *Piaractus mesopotamicus* [30], a tropical fish with great commercial importance in Brazilian aquaculture [31], submitted to prolonged fasting followed by refeeding. The proteomics analysis suggested the differential expression of a particular protein, the PVALB, so in the second step of this manuscript we tested the hypothesis that the PVALB protein and the *pvalb* gene are differentially expressed in the fast-twitch muscle of fasted and refed fish.

## Material and methods

### Ethics statement

All experiments were performed in accordance with the Ethical Principles in Animal Research adopted by the Brazilian College of Animal Experimentation (COBEA). This protocol was approved by the Ethics Committee on Animal Use (CEUA, Protocol number 721), Institute of Biosciences of Botucatu, São Paulo State University, Botucatu, São Paulo, Brazil.

The welfare of fish was confirmed by analysing the overall appearance of animals and was assured by maintaining proper oxygenation, temperature, and ammonium levels. The period of fasting was prolonged but not exaggerated, considering that other studies involving fish use equal or longer food restriction periods [32–35].

### Fish rearing, fasting and refeeding treatments

The fish rearing procedures and diet treatments were previously described by Gabriel Kuniyoshi et al. [16]. Pacus were obtained from São Paulo Agency for Agribusiness Technology (APTA), Presidente Prudente, São Paulo, Brazil. The animals were maintained with the environmental parameters used for this species in aquaculture. Juvenile fish (~100 g) were reared at 28˚C under natural photoperiod (12 hours of light: 12 hours of dark) in storage tanks of 0.5m^3^ equipped with separate systems of water circulation. The dissolved oxygen levels were monitored daily, while the levels of ammonium, nitrite, and nitrate were monitored weekly. The wellness of fish was availed by observing their overall appearance. The foodstuff used in this study was the commercial fish food Guabi-Pirá (extruded, 28% protein, 225 mg∙kg^-1^ vitamin C, 6–8 mm diameter). The sex of the fish was not determined, since this species does not reproduce in captivity unless hormone treatment is given [36], and thus the reproductive cycle could not affect our results.

Fish were acclimatized for 1 week under satiety feeding conditions before the onset of the diet treatments. The animals were separated in two groups: Control group (C), which was normally fed for 60 days, and Experimental group (E), which was fasted for 30 days and refed for 30 days. There were three replicate tanks per group, and each of them was considered an experimental unity. Each tank contained 24 fish at the onset of the experiment.

After fasting and during refeeding, fish were euthanized with benzocaine in a concentration of approximately 250 mg∙L^-1^ [37], and the body weight and standard length were measured (n = 9 for each group/time point). Samples of fast-twitch muscle were collected from the epaxial region, after 30 days of fasting and after 6, 24, 48 hours and 30 days of refeeding (n = 9 for each group/time point). The samples were frozen in liquid nitrogen for subsequent analysis.

### Protein extraction

Sarcoplasmic protein was extracted from the fast-twitch muscle of C and E groups after 30 days of fasting and after 30 days of refeeding (n = 9 for each group/time point). Protein extraction was carried out by homogenizing 5 g of sample in 10 mL of 10 mM Tris–HCl buffer (pH 7.2) with 5 mM Phenylmethanesulfonyl fluoride (PMSF), 8M Urea and 65 mM Dithiothreitol (DTT). The resulting suspension was vortexed for 2–3 min and centrifuged for 15 min at 9,690 g and 4°C. The supernatant, which contained the sarcoplasmic protein fraction, was recovered and quantified by Bradford assay using BSA standard [38].

Samples were mixed into protein pools. Each pool contained proteins from three different fish. There were three pools for each group at each collection point.

### In solution digestion of proteins

The sarcoplasmic proteins (50μg) were digested in a solution of 8 M urea (1:1) followed by 25 minutes of incubation with 10 mM DTT at 56°C. Alkylation was performed by adding 14 mM iodoacetamide (IAA) during 30 minutes at room temperature, avoiding light exposure. To quench free IAA, samples were reincubated for 15 minutes with 5 mM DTT, and subsequently, 1 mM calcium chloride was added to the samples. The hydrolysis occurred in the presence of trypsin enzyme (Promega, Madison, WI) (1:50 enzyme/substrate) for 18 hours at 37°C. Trypsin was inactivated with 0.4% formic acid solution. The samples were desalinated using Sep-Pak Vac C18 cartridges (Waters, Milford, MA, USA). The peptide fragments were reduced with vacuum centrifuge and kept at -20°C until analysis by mass spectrometry.

### Liquid chromatography-tandem mass spectrometry

During our experiments, the protein pools (n = 3 pools containing proteins from 3 animals each) were run in triplicate. Liquid chromatography-tandem mass spectrometry (LC-MS/MS) was performed in a Q-Tof PREMIER API mass spectrometer (MicroMass/Waters, Milford, MA) at the Brazilian Biosciences National Laboratory (LNBio), Campinas, Sao Paulo, Brazil. An aliquot of 4.5 μL containing 5μg of peptides was separated by C18 (100 um x 100 mm) RP-nanoUPLC (nanoAcquity, Waters) coupled with a Q-Tof Premier mass spectrometer (Waters) with nanoelectrospray source at a flow rate of 0.600 μl/min. The gradient was 2–90% acetonitrile in 0.1% formic acid over 60 minutes. The nanoelectrospray voltage was set to 3.5 kV, a cone voltage of 30 V and the source temperature was 80ºC. The instrument was operated in the ‘top three’ mode, in which one MS spectrum is acquired followed by MS/MS of the top three most-intense peaks detected. After MS/MS fragmentation, the ion was placed on the exclusion list for 60 s and a real-time exclusion was used. The spectra were acquired using software MassLynx v.4.1 and the raw data files were converted to a peak list format (mgf) without summing the scans by the software Mascot Distiller v.2.3.2.0, 2009 (Matrix Science Ltd.) and searched against Actinopterygii taxonomy NCBI database (May, 2017; 1,939,396 sequences; 936,617,147 residues) using Mascot engine v.2.3.01 (Matrix Science Ltd.), with carbamidomethylation as fixed modifications, oxidation of methionine as variable modification, one trypsin missed cleavage and a tolerance of 0.1 Da for both precursor and fragment ions.

The mass spectrometry proteomics data have been deposited to the ProteomeXchange Consortium via the PRIDE [39] partner repository with the dataset identifier PXD014232.

### Bioinformatics analysis of proteomic data

For the relative protein quantification between C and E groups, data from Mascot were analyzed using Scaffold Q+ software Version 4.3.4 (Proteome Software Inc, Portland, USA). The label-free quantitation method reports the differentially expressed proteins without the use of a stable isotope or tag. The spectral counting counts refer to the number of spectra identified for a given peptide. Scaffold calculates the fold change difference in terms of spectrum counts of each protein. For a given protein, the closer the fold change value is to 1, the less it is differentially expressed. The proteins with fold change ≥1.5 or ≤0.6 were considerately differently expressed between groups.

The protein interaction network was obtained using the software STRING (https://string-db.org/) [40] with minimum interaction score of medium confidence. Based on our STRING results, 3D protein interaction networks were obtained with the software MoNet (Biocomplexity laboratories, https://www.biocomplexitylab.net/en/softwares/monet/), with confidence of 60%, reference organism as *Danio rerio* and “coexpression”, “experimental” and “database” as interaction sources.

### Western blot analysis

For the Western blot analysis, unpolled protein samples were used (n = 6). Lammeli buffer (Sigma, USA) were added to extracted proteins from C and E groups and boiled at 100°C for 10 minutes. Next, proteins samples were separated according to molecular weight with SDS-PAGE in 15% polyacrylamide gels, for 140 minutes. After electrophoresis, proteins were electro-transferred to nitrocellulose membranes (Bio-Rad, USA). The blotted membranes were blocked with 5% nonfat dry milk in TBS buffer containing 0.5% Tween 20 (TBST) for 2 hours at room temperature, and then incubated overnight at 4–8°C with specific antibodies against PVALB (PARV-19, P3088, Sigma Aldrich, USA; 1:2000 dilution) [41,42] and β-actin (Santa Cruz Biotechnology, USA; 1:200 dilution). Binding of the primary antibody was detected with peroxidase-conjugated secondary antibodies (rabbit or mouse, depending on the protein, for 2 hours at room temperature), using enhanced chemiluminescence (Amersham Biosciences, USA), and detected by autoradiography in ImageQuant^™^ LAS 4000 (GE Healthcare, USA). Quantification analyses of blots were performed with ImageJ software. Targeted bands were normalized to the expression of β-actin.

### Gene expression analysis

For mRNA analysis, total RNA was extracted from fast-twitch muscle samples in the C and E groups using TRIzol Reagent (Life Technologies, USA), according to the manufacturer’s recommendations. RNA quantification was performed using the spectrophotometer NanoVue Plus (GE Healthcare, USA). The integrity and purity of the extracted RNA were assessed with agarose electrophoresis. Samples with low quality were not included in further analyses. Extracted RNA was treated with DNase I Amplification Grade (Life Technologies, USA) to eliminate any possible contamination with genomic DNA from the samples. mRNA reverse transcription was performed using the High-Capacity cDNA Reverse Transcription Kit (Applied Biosystems, USA), following the manufacturer’s guidelines.

The expression levels of mRNAs were assessed by quantitative real-time PCR (RT-qPCR) using QuantStudio^™^ 12K Flex Real-Time PCR System (Life Technologies, USA), according to a protocol adapted from works previously published by our research group [13,43]. The cDNA was amplified by GoTaq qPCR Master Mix (Promega, USA). Primers were designed using Geneious version 4.8 (https://www.geneious.com/) and Primer Express 3.0.11 (Life Technologies, USA) according to data available for pacu skeletal muscle transcriptome from the European Nucleotide Archive (PRJEB6656) [44] (S1 Table), and were synthesized by Thermo Fisher (USA). The expression levels were normalized by *rpl13*, whose expression was constant among all muscle samples. The relative quantification of gene expression was performed by the comparative Ct method [45].

### miRNA prediction

We used a combination of bioinformatic tools to identify miRNAs that possibly control parvalbumin expression. First, we searched the gene target “*pvalb*” in miRWalk Version 3, against human, mouse and rat databases on June 1st of 2019 (http://mirwalk.umm.uni-heidelberg.de/) [46]. We searched against these species databases due to the lack of information about *Piaractus mesopotamicus* in miRWalk. The resulting miRNAs and target RNAs lists were used to create a Venn diagram with the VIB—Genth University Venn diagram maker available at http://bioinformatics.psb.ugent.be/webtools/Venn/. The miRNAs with most target RNAs were selected for further analysis with BibBiServ RNAhybrid, which was used for finding the minimum free energy of the miRNA and target RNA hybridization (https://bibiserv.cebitec.uni-bielefeld.de/rnahybrid) [47]. For RNAhybrid analysis, the target was the 3’ UTR *pvalb2* gene of zebra fish (*Danio rerio*). The target RNA secondary structure was built with RNAstructure Web Server using the sequence of the 3’ UTR of the *Danio rerio pvalb2* gene (https://rna.urmc.rochester.edu/RNAstructureWeb/) [48].

### Statistical analyses

In this manuscript, body weight is expressed as a mean ± confidence interval. The statistical comparison of absolute body weight between C and E groups was performed previously by Gabriel Kuniyoshi et al [16].

The relative rate of increase (RRI) was calculated with the mean, standard length and mean body weight, according to Eqs 1 and 2, respectively [49]. As means were used to calculate RRI, its standard deviation could not be calculated.

$${RRI}_{length}=100*\frac{Finall\;ength-Initial\;length}{Initial\;length}$$ (1)

$${RRI}_{weight}=100*\frac{Final\;weight-Initial\;weight}{Initial\;weight}$$ (2)

For the RT-qPCR analysis, outliers were eliminated with the method of median absolute deviation (MAD) [50]. After 6 and 24 hours of refeeding, the E group samples were compared with the C group samples collected after 30 days of fasting, since it was not possible to slaughter C group animals after 6h and 24h of refeeding. At the other time points, we had measurements for both the E and C groups. Mann-Whitney test was used to compare mRNA relative expression between C and E groups (p < 0.05). For Western Blot results, lanes affected by bubbles were eliminated from analysis, and the means of C and E groups were compared with unpaired t-test (p < 0.05).

## Results

### Growth parameters and gene expression

In our experiment, we compared fish fasted for 30 days and refed for 30 days (E group) to regularly fed fish (C group). To assess the presence of growth and atrophy during our treatment, we measured RRI_length_ and RRI_weight_, as well as *igf-1* and *mafbx* gene expression in fast-twitch muscle.

**Fig 1:**
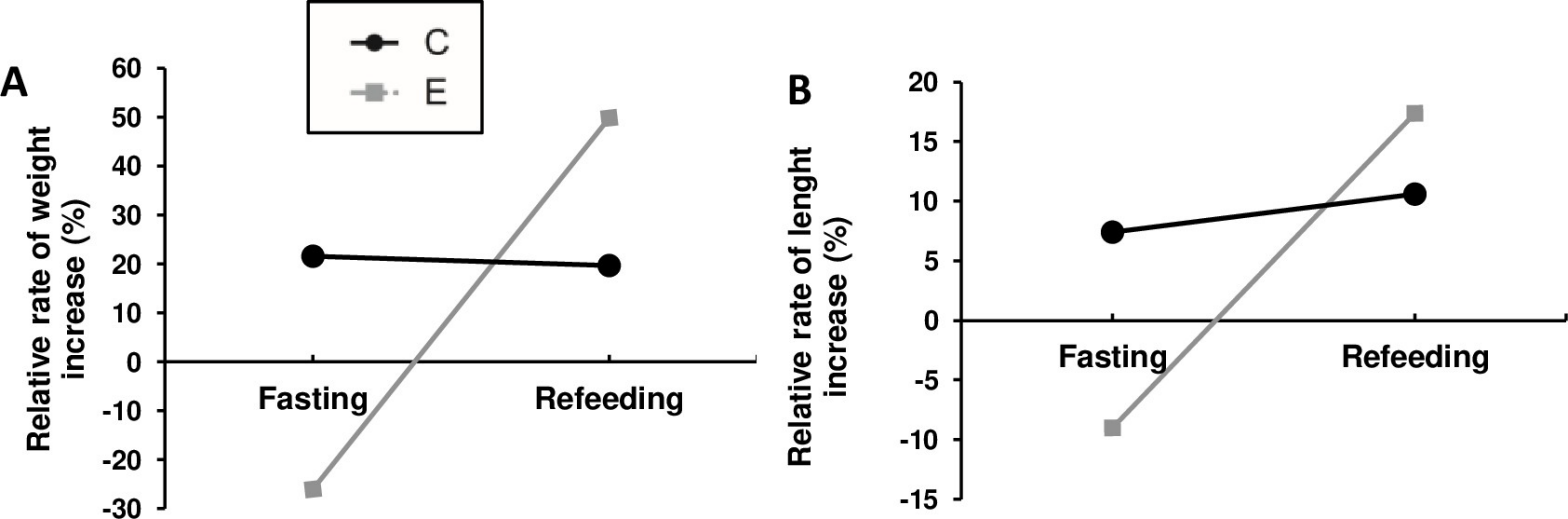
Relative rate of weight and length increase of juvenile pacu after fasting and refeeding. **A)** Relative rate of weight increase (RRI_weight_) after 30 days of fasting and 30 days of refeeding. **B)** Relative rate of length increase (RRI_length_) after 30 days of fasting and 30 days of refeeding. C = control Group; E = experimental group.

**Fig 2:**
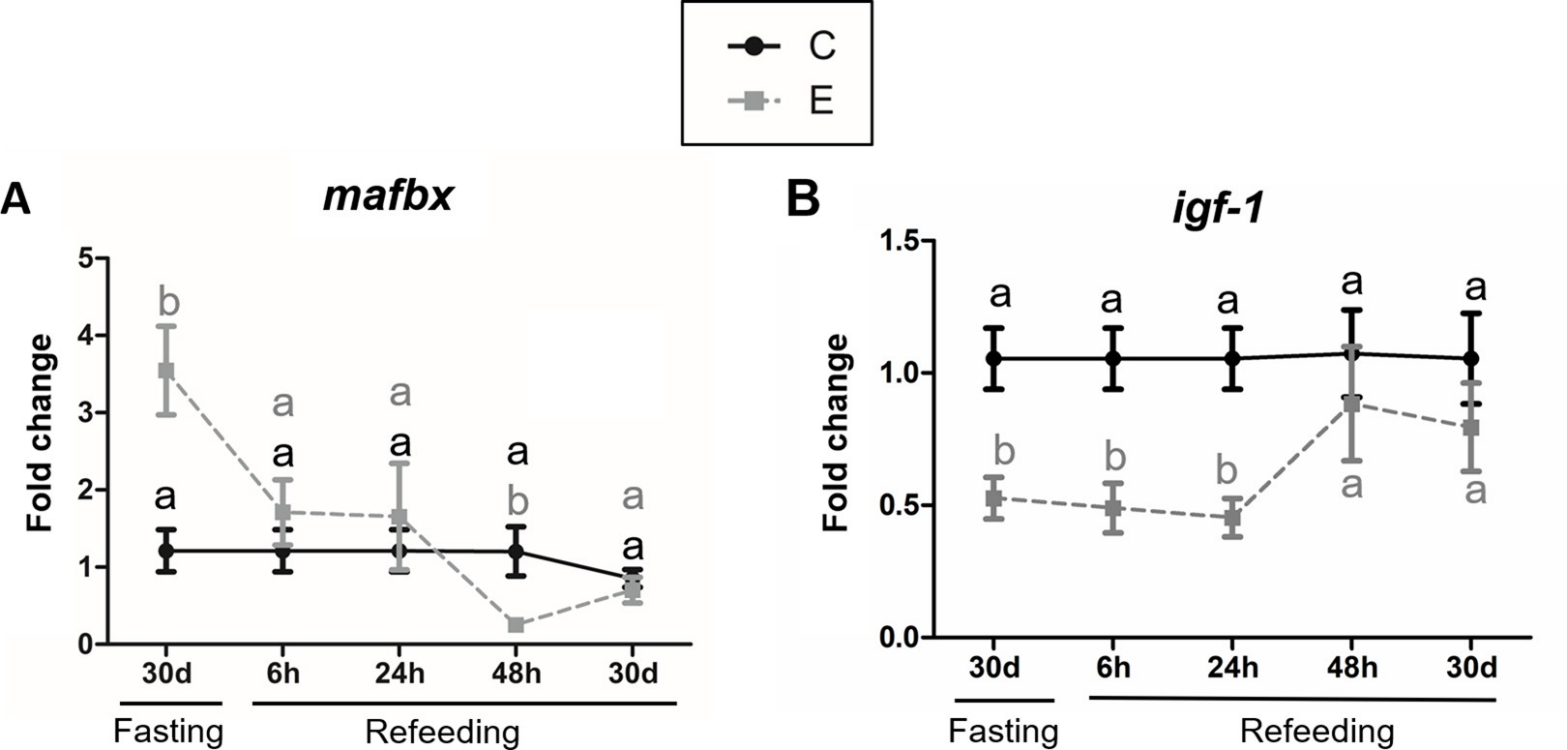
Catabolic and anabolic gene expression in fast-twitch muscle of juvenile pacu after fasting and refeeding. **A)** Gene expression of the catabolic gene *mafbx* after 30 days of fasting and 6h, 24h, 48h and 30 days of refeeding. **B)** Gene expression of the anabolic gene *igf-1* after 30 days of fasting and 6h, 24h, 48h and 30 days of refeeding. The gene expression analysis was performed with RT-qPCR. Gene expression is relative to the control *rpl13*. Different letters indicate a significant difference between C and E in the Mann-Whitney test (α = 0.05). Results are expressed as a mean ± SEM. C = control Group; E = experimental group.

After prolonged fasting, group E (weight = 80.81±14.48 g) presented decreased RRI_length_ and RRI_weight_ in comparison with group C (weight = 132.77±32.48g) (Fig 1A and 1B, S2 and S3 Tables). Fasting led to an increase in *mafbx* gene expression and a decrease in *igf-1* gene expression in E group (Fig 2A and 2B, S4 Table).

After refeeding, E group (weight = 121.18±19.65g) presented a greater RRI_length_ and RRI_weight_ than C group (weight = 158.97±20.73g), without fully recovering weight (Fig 1A and 1B, S2 and S3 Tables), which represents partial compensatory growth [14]. The gene expression of *mafbx* decreased after refeeding, being similar to C group after 6h and 24h, and downregulated at 48h (Fig 2A, S4 Table). The gene expression of *igf-1* was decreased after 6h and 24h and showed no differences at 48h (Fig 2B, S4 Table). There was no difference in *igf-1* and *mafbx* levels after 30 days of refeeding (Fig 2A and 2B, S4 Table).

### Shotgun proteomics

**Fig 3:**
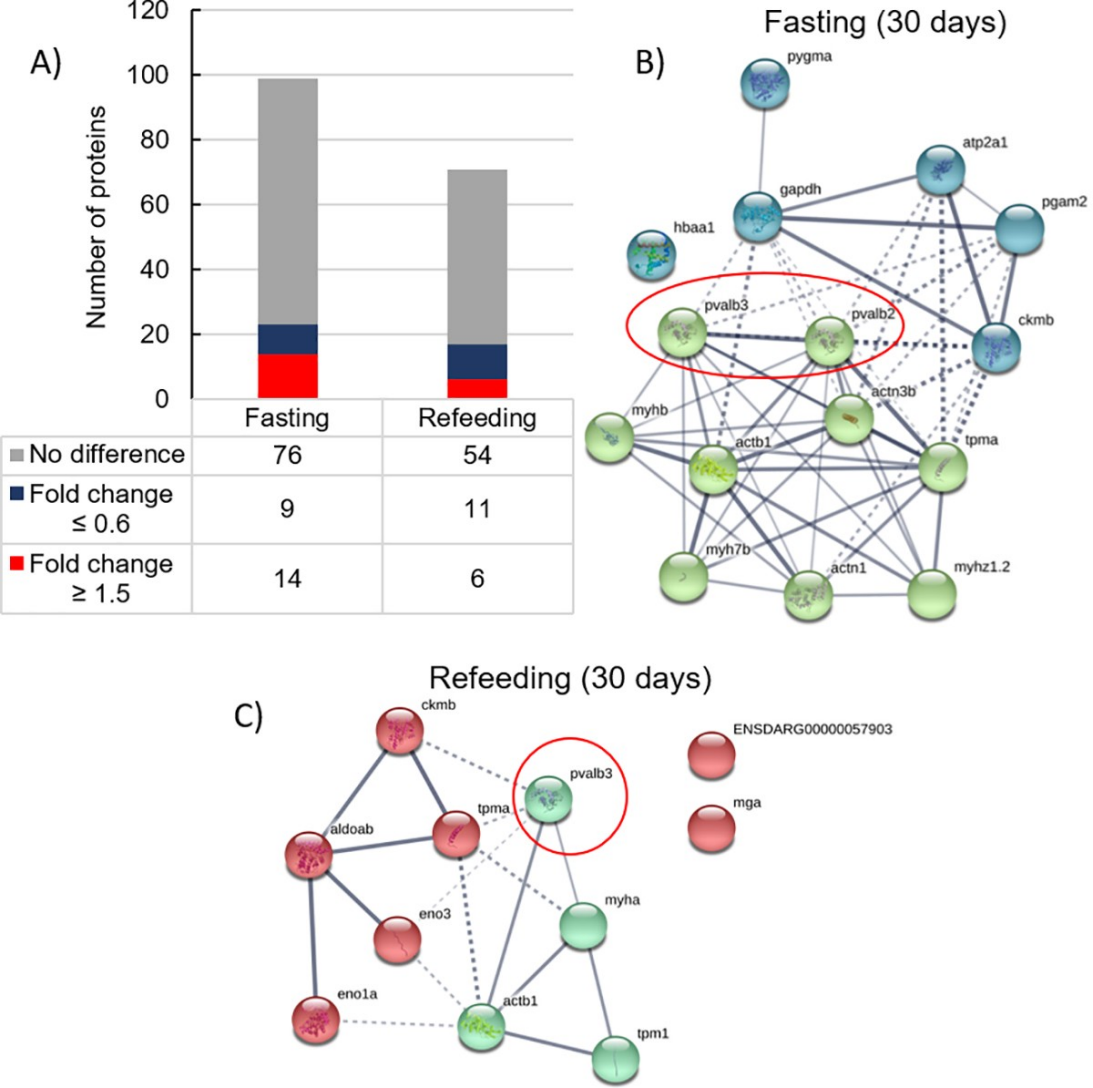
Shotgun proteomics of fast-twitch muscle of juvenile pacu after fasting and refeeding. **A)** Number of identified proteins with fold change ≥1.5 (upregulated), ≤0.6 (downregulated) or with fold-changed between 0.6 and 1.5 (unchanged) after 30 days of fasting and 30 days of refeeding. **B-C)** Protein interaction networks made with STRING software. Each node represents a protein. The lines connecting the nodes represent a possible interaction between the two proteins. The thickness of the lines represents the confidence of interaction between the proteins. The PVALB is highlighted by red circles. **B)** Network of the proteins differentially expressed after 30 days of fasting. Metabolism proteins as blue nodes and cytoskeleton/muscle contraction proteins as olive nodes. **C)** Network of the proteins differentially expressed after 30 days of refeeding. Cytoskeleton/muscle contraction proteins as green nodes and metabolism proteins as red nodes.

The shotgun proteomics analysis in fast-twitch muscle revealed 99 proteins after fasting (S5 Table). After prolonged fasting, 23 proteins were differentially expressed, being 14 upregulated and 9 downregulated (Fig 3A). The protein interaction network showed proteins related to cytoskeleton, muscle contraction, and glucose metabolism (Fig 3B, S1 Video).

After refeeding, a total of 71 proteins were identified (S6 Table). There were 17 differentially expressed proteins, 6 upregulated and 11 downregulated. The protein interaction network indicated that refeeding was also characterized by a change in metabolic and muscle contraction proteins (Fig 3C, S2 Video).

Both after 30 days of fasting and 30 days of refeeding, the proteomic analysis showed downregulation of some isoforms of PVALB. After 30 days of fasting, the isoforms ACP30426.1 and KPP74939.1 had fold changes of, respectively, 0.5 and 0.6, whilst after 30 days of fasting the isoform P09227.2 had a fold change of 0.5. This finding was key to elaborate the hypothesis of parvalbumin gene and protein regulation in the muscle of fasted and refed fish.

### Parvalbumin expression

**Fig 4:**
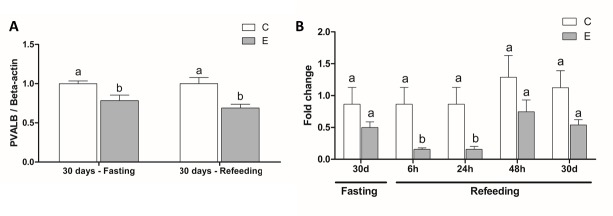
Parvalbumin gene and protein expression in fast-twitch muscle of juvenile pacu after fasting and refeeding. **A)** Western blot analysis of PVALB protein expression after 30 days of fasting and 30 days of refeeding. β-actin was used for normalization. Different letters indicate significant difference between C and E in t-test (α = 0.05) **B)** RT-qPCR analysis of *pvalb* gene expression after 30 days of fasting and 6h, 24h, 48h and 30 days of refeeding. The *rpl13* gene was used for normalization. Different letters indicate a significant difference between C and E in Mann-Whitney test (α = 0.05). Results are expressed as a mean ± SEM. C = control Group; E = experimental group.

The Western Blot analysis confirmed that PVALB protein expression was downregulated both after fasting and after refeeding (Fig 4A, S1 Fig, S7 Table). In the RT-qPCR gene expression analysis, the statistical analysis only indicated differential expression after 6h and 24h of refeeding, and there was no statistical difference in *pvalb* gene expression after 30 days of fasting and after 48h and 30 days of refeeding (Fig 4B, S4 Table).

### miRNA prediction

Considering that the *pvalb* mRNA expression levels were not changed and that the PVALB protein levels were decreased, we sought to identify the microRNAs that potentially regulate the parvalbumin expression. The miRWalk searches returned results regarding five different *pvalb* transcripts, two in the human database (NM_001315532, NM_002854), two in the mouse database (NM_013645, XM_006520643) and one in the rat database (NM_022499). We found 444 unique miRNAs that were predicted to target these *pvalb* transcripts (Table 1 and S8, S9, S10 and S11 Tables).

**Table 1:** miRWalk search results. Number of predicted miRNAs that potentially target the *pvalb* transcripts registered at the miRWalk human, mouse and rat databases.

Species	RefSeq ID	Number of predicted miRs	Number of unique predicted miRs
**Human**	NM_001315532	173	158
**Human**	NM_002854	158	147
**Mouse**	NM_013645	275	253
**Mouse**	XM_006520643	90	86
**Rat**	NM_022499	33	30
**Total of unique predicted miRs**			444

**Fig 5:**
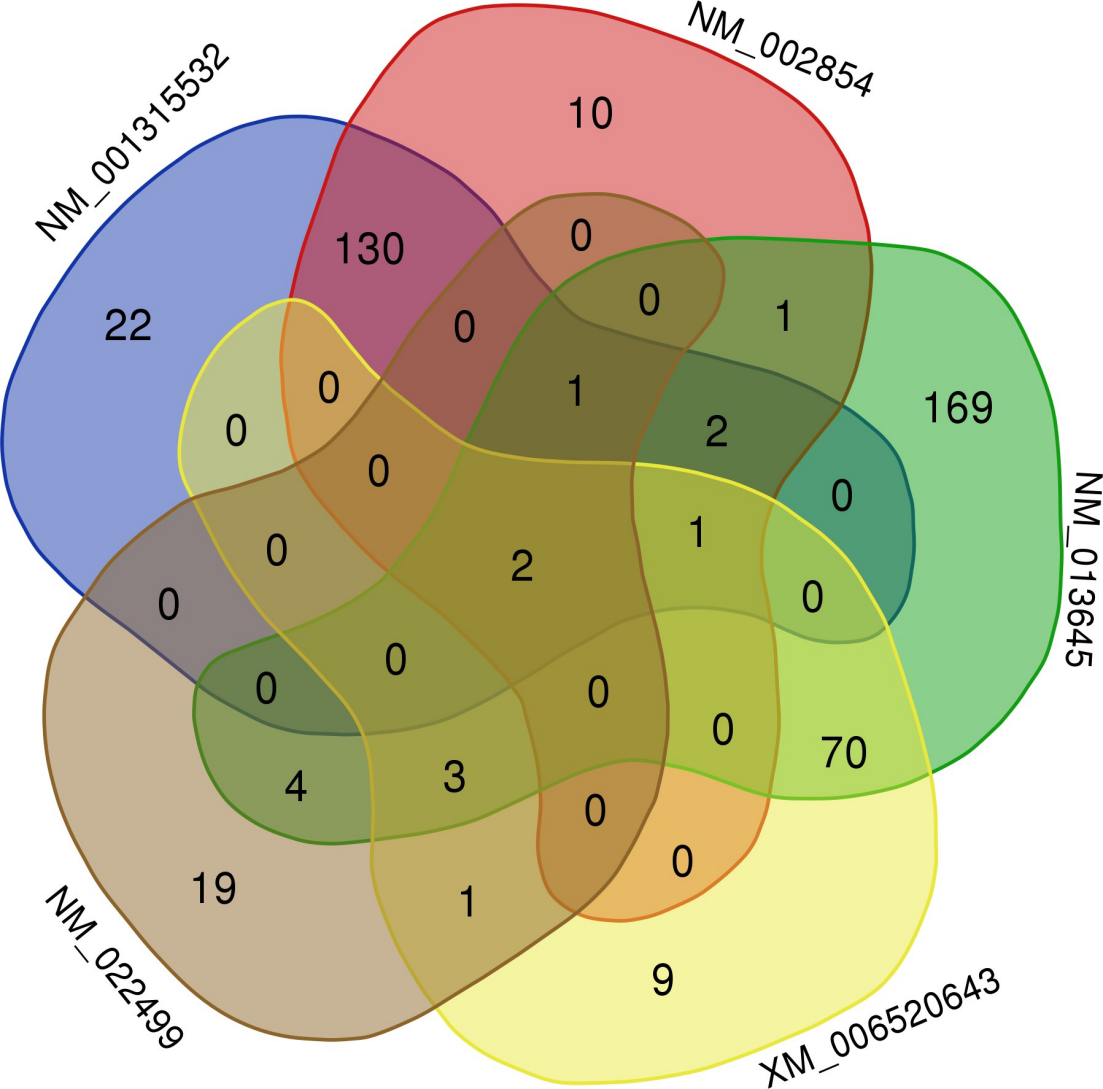
Venn diagram of the number of miRNAs targeting each *pvalb* transcript. Each group represents a *pvalb* transcript. The miRNA analysis was performed with miRWalk and the Venn diagram was designed with the Genth University online tool available at http://bioinformatics.psb.ugent.be/webtools/Venn/.

The Venn diagram analysis showed that two miRNAs, miR-23a-5p and miR-149-3p, were predicted to regulate all the five *pvalb* transcripts (Fig 5 and S11 Table).

The hybridization of miR-23a-5p with *pvalb2* 3’ UTR presents a minimum free energy of -26.5 kcal/mol (S2 Fig). The hybridization of miR-149-3p with *pvalb2* 3’ UTR present a minimum free energy of -26.6 kcal/mol (S3 Fig). Since that minimum free energy is an important predictor of strong interaction between RNA molecules [47], the values found for miR-23a-5p with *pvalb2* 3’ UTR and for miR-149-3p with *pvalb2* 3’ UTR suggest a strong interaction between microRNAs and pvalb transcripts.

## Discussion

Low or no food availability is often found in the wild and probably selected mechanisms of acclimation to fasting in fish since these animals can withstand long periods without food [51]. In addition, fish may present accelerated growth upon reestablishment of feeding, a mechanism known as compensatory growth [14]. In both scenarios of fasting and refeeding, there is intense muscle plasticity, which involves well-known components such as the atrogenes *mafbx* and *murf1*, which promote muscle catabolism [4], and the growth factor *igf-1*, related with muscle anabolism [6]. However, additional mechanisms controlling muscle anabolic and catabolic pathways are present. In this paper, we adopted proteomic approaches to explore the molecular changes caused by prolonged fasting and refeeding in fish muscle.

After prolonged fasting, the decrease in RRI, the upregulation of *mafbx*, and the downregulation of *igf-1* suggest increased catabolism and decreased anabolism in pacu muscle. The upregulation of *mafbx* may be associated to the downregulation of some sarcomere proteins in our proteomic analysis, since the degradation of these proteins may have provided an energetic substrate for the maintenance of cellular activities during fasting [52,53]. The downregulation of *igf-1* gene may be related to the interruption of muscle growth upon lack of nutrients, and also to the activation of catabolic pathways, since *igf-1* inhibits atrogenes activity [54]. These results support the work of earlier studies [12,13,16,55] and suggest the occurrence of muscle atrophy during prolonged fasting.

After short refeeding (6h-24h of refeeding), the expression of mafbx was similar to C group while the *igf-1* remained downregulated. Perhaps the *mafbx* returned to its basal levels because the nutrients sources were once again available and *igf-1* was still downregulated because muscle growth is only resumed when the body reserves are restored [14], and maybe it was not complete after short refeeding. This is in accordance with previous studies that show a decrease in atrogenes expression at the beginning of the refeeding period [13,56] and upregulation of *igf-1* only on late refeeding [56].

After 48h of refeeding, there was an increase in *igf-1* and a decrease in *mafbx* expression. This may mark the moment where anabolism surpasses catabolism, resulting in muscle re-growth. After this period, at 30 days of refeeding, fish presented partial compensatory growth and no differential expression of *mafbx* and *igf-1* genes. Taken together, our results suggest the restoration of muscle homeostasis from 48h of refeeding.

Our proteomic analysis identified 99 proteins after fasting and 71 proteins after refeeding, summing 170 protein identifications. At a certain degree, the number of identifications matches those of earlier studies, such as Carrera et al. [57] who found 183 non-redundant proteins in fish sarcoplasm by using shotgun proteomics. However, the number of proteins identified in our work was smaller than in shotgun analyses of well-stablished models, such as *Homo sapiens*. For instance, in a shotgun proteomics analysis of human skeletal muscle, more than 2000 proteins were identified [58]. This discrepancy is found because, unlike well-known models, *P*. *mesopotamicus* does not have the genome sequenced, so our proteins identification had to be homology-driven, which results in fewer identifications, especially regarding non-conserved proteins [26]. This explains the low protein identification when compared to proteomic analyses of classic animal models.

Several of the identified proteins in our study were differentially expressed after prolonged fasting and refeeding condition. Most of the differentially expressed proteins were involved with the cytoskeleton, muscle contraction, and glucose metabolism. This may be due to the sarcomere remodeling during muscle catabolism and re-growth, and to the modifications in metabolism upon changes in diet [13,59]. For instance, in our study, the upregulation of glycogen phosphorylase (pygma, AAH95379.1) after fasting may be related to the recovery of glucose from glycogen reserves during food shortage [60]. We also identified differential expression of myosin isoforms in our study (up or down-regulated), which were already found to be changed in fasted/refed fish muscle [15,52]. Moreover, after fasting, we observed downregulation of Cofilin-2 (XP_007240534.1), which is involved in actin depolymerization and apoptosis [61]. Although the changed abundance of some of these proteins was already described by previous studies, this is the first time that some factors, such as PVALB, were described as part of fast-twitch muscle response to fasting and refeeding, and this is key for the advances of muscle proteostasis research.

The changed pathways in the present shotgun analysis match those found to be affected in our previous gel-based proteomic analysis [16]. This corroboration between the two publications shows that their conclusions are robust, since two different proteomic approaches, published separately, showed similar results. However, many of the differentially expressed proteins were identified in one study but not in another, which may be due to differences in the sensitivity and resolution of the two techniques [28], and because only some protein spots were characterized in the previous study [16]. This shows that shotgun proteomics (present study) and two-dimensional proteomics (previous study, [16]) are complementary and that the use of both gives us a more complete view of the changes caused by prolonged fasting and refeeding conditions.

In proteomics, isoforms are variants of a protein that present akin, but distinct, peptide sequences. Isoforms can be encoded by separate genes, or they can be encoded by the same gene and differ due to alternative splicing and post-translation modifications [62]. Interestingly, in our present proteomic analysis, isoforms had distinct fold changes even though they were variations of the same protein. This happens because isoforms frequently present distinct functions and cellular location, and thus different expression patterns [62]. For instance, our results show that after fasting an isoform of alpha-tropomyosin was upregulated (XP_014031659), while others were not (XP_007239611, XP_007246561, XP_014004080, etc.), reflecting the functional and compartmental diversity of tropomyosin isoforms [63]. Vareilles et al [64], who analyzed proteomics of fish larvae, found alpha-actin to be up or down-regulated, depending on the isoform, and used a hypothesis similar to ours to explain the variability. An alternative explanation lies on the methodology of shotgun proteomics: as proteins are digested prior to mass spectrometry, the sequences that differentiate isoforms are more difficult to detect and problems with the identification of variants may occur. Furthermore, databases often have protein redundancy, leading to false identification of isoforms [62]. However, these technical issues are reduced in bottom-up proteomics. As a previous bottom-up analysis of similar samples also showed differential expression of isoforms [16], we assume that the correct explanation is that distinct expression pattern is due to distinct localization and function.

Among the proteins dysregulated in the condition evaluated, we selected PVALB for additional gene and protein expression validation for several reasons. First, PVALB is a seafood cross-reactive allergen that is linked to 90% of fish allergy cases [65]. Secondly, PVALB is related to Ca^2+^ transportation inside the cells, and Ca^2+^ is an important second messenger in various pathways, including muscle plasticity [25]. Lastly, PVALB was never analyzed in such treatment before: even though it was already identified in proteome of fish [66–68], and studied in the muscle of mammals [69,70], we have found no research that investigate PVALB in muscle of fish submitted to fasting and refeeding.

After 6h and 24h of refeeding, the *pvalb* gene expression was downregulated. Possibly, this indicates the involvement of parvalbumin in the early response to fasting, thus accompanying the downregulation of *igf-1* and the drop in *mafbx* levels. Although we could not provide the protein expression analyses of muscle after 6h, 24h and 48h of refeeding we believe that the PVALB protein expression was downregulated at these time points, considering that it was decreased both at the beginning and at the end of the fasting period.

**Fig 6:**
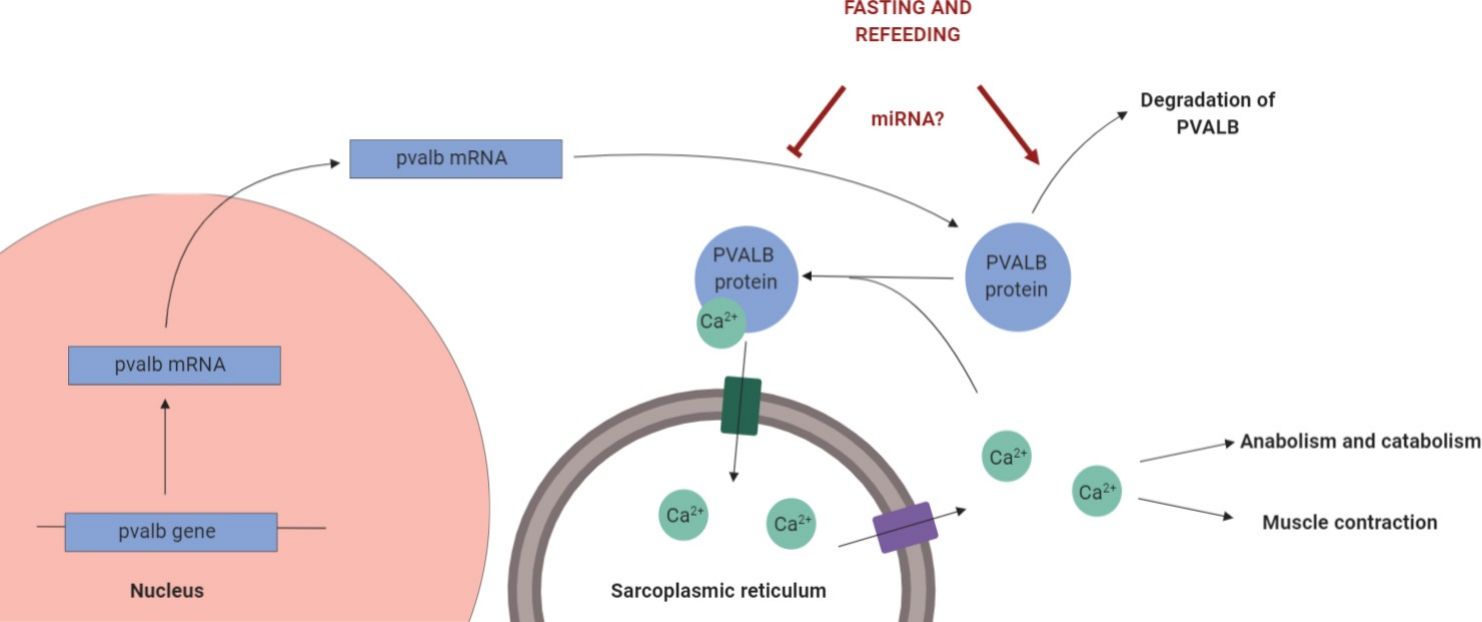
Model of *pvalb* regulation and its effects in the fast-twitch muscle of fasted and refed fish. According to previous literature, the PVALB protein translocates calcium from the cytoplasm to the sarcoplasmic reticulum, what can have implications in muscle contraction and catabolic and anabolic processes. In the present work, we show that the fasting and refeeding affects post-transcriptionally the parvalbumin expression, possibly though a miRNA.

Our results show that, after 30 days of fasting and 30 days of refeeding, there is downregulation of PVALB protein, but not of the *pvalb* gene, thus refutating our initial hypothesis. However, this launches a new hypothesis: there may be a post-transcriptional regulation of parvalbumin that inhibits its translation or increases its protein degradation (Fig 6). A type of post-transcriptional regulatory element that may be involved in parvalbumin regulation are the microRNAs (miRNAs). The miRNAs are short non-coding RNAs that usually bind to the 3’ UTR region of the target mRNA and prevent its translation or promotes its degradation [71,72]. As a matter of fact, miRNAs play an important role in the skeletal muscle of fish [13,43,72]. For this reason, we used bioinformatics to predict miRNAs that may control parvalbumin expression in model species, and found almost 450 candidates. Two miRNAs may target all the studied parvalbumin transcripts: miR-23a-5p and miR-149-3p, both with minimum free energy of -26 kcal/mol. We do not have enough evidence that these miRNAs are responsible for downregulation of parvalbumin in fish skeletal muscle, but they are interesting candidates that can be studied in future research.

While the miRNAs are a possible cause of parvalbumin regulation, it is also interesting to ponder the consequences of PVALB differential expression in fish muscle. The differential expression of PVALB may be related to changes in Ca^2+^ cytoplasmic levels since PVALB translocates the ion to the sarcoplasmic reticulum (Fig 6). As a matter of fact, in our proteomic analysis, there was changed abundance of SERCA. SERCA is a pump that transports Ca^2+^ from the cytoplasm to the sarcoplasmic reticulum [25], so its differential expression is another evidence of altered Ca^2+^ homeostasis. The changes in Ca^2+^ location may have implications in muscle anabolic and catabolic pathways. For example, Ca^2+^ activates the protease calpain, which promotes degradation of some muscle structural proteins [73,74]. Also, cytoplasmic Ca^2+^ may be related to muscle growth and differentiation [5,25].For example, myotubes respond *to igf-*1 with a IP3-mediated increase in Ca^2+^ concentration [75]. Also, the muscle cells response to insulin involve increase of near-membrane free Ca^2+^ concentration, probably by influx of extracellular Ca^2+^ [76]. In addition, extracellular Ca^2+^ influx and the calmodulin-Ca^2+^ pathway are involved in satellite cells activation [77]. This does open doors for additional studies about a possible role of PVALB in controlling muscle plasticity through regulation of Ca^2+^.

Taking this into consideration, we believe that PVALB may be involved in the process of response to fasting and refeeding. In mammals, decreased abundance of PVALB is related to muscle loss caused by ovariectomy, and increased levels of PVALB are related to restoration of muscle after estrogen replacement [69]. Other studies with mammals have shown that PVALB levels in muscle are reduced in senescence-related sarcopenia and increased after exercise [70]. The findings of the present work indicate that PVALB may also be related to muscle atrophy and re-growth during fasting and refeeding condition in fish. This way, PVALB may be a biomarker of long-term fasting and refeeding.

In summary, the critical interpretation of the phenomena discovered in our research permitted the development of many interesting hypotheses, specially about the causes and consequences of PVALB differential expression. These hypotheses, despite being based on previously published literature and in our results, must be further validated by future research; nonetheless, this does not diminish our manuscript, since it has opened doors for these prospective works. For example, the hypothesis of PVALB being involved in the response to fasting could be tested by measuring the levels of atrophic and growth factors in cultured fish myocytes genetically engineered to down-express PVALB, in a media with low nutrient availability. The hypothesis of altered Ca^+^ dynamics could be studied with patch-clamp and Ca^+^ imaging techniques. We did not perform these analyses in the current paper because our aim was to present a holistic view of skeletal muscle and to be a basis for upcoming works.

## Conclusions

In conclusion, our results showed that prolonged fasting followed by refeeding promoted changes in fast-twitch muscle proteome, especially in proteins involved with cytoskeleton, muscle contraction, and metabolic process. PVALB protein was downregulated in fast-twitch muscle of fasted and refed fish. This fact may characterize this protein as an important factor expressed during prolonged fasting followed by refeeding in fish muscle, which is an information that may be useful in aquaculture studies. This study was the first shotgun proteomic analysis of fast-twitch muscle during prolonged fasting followed by refeeding, and the first study on parvalbumin in this condition.

## Supporting information

S1 Table Sequence of the primers used for quantitative real-time PCR (RT-qPCR) genes amplification in juveniles pacu (*P*. *mesopotamicus*) fast-twitch muscle. F–Forward sequence; R–Reverse sequence.
(DOCX)

S2 Table Descriptive data of weight (grams) of *Piaractus mesopotamicus* submitted to 30 days of fasting and 30 days of refeeding. (DOCX)

S3 Table Descriptive data of length (centimeters) of *Piaractus mesopotamicus* submitted to 30 days of fasting and 30 days of refeeding. (DOCX)

S4 Table Fold-change of mafbx, igf-1 and pvalb in the fast-twitch muscle of *Piaractus mesopotamicus* after 30 days of fasting and 6h, 24h, 48h and 30 days of refeeding. Gene expression was assessed with RT-qPCR. The fold-change was calculated by the comparative Ct method and is relative to the control rpl13. The same control was used for 6h and 24h of refeeding in order to minimize the number of slaughtered animals.
(DOCX)

S5 Table Identification of 99 proteins by shotgun proteomic in pacu fast-twitch muscle after 30 days of fasting. Proteins were considered differently expressed between group C and E when fold change ≥1.5 or ≤0.6.
(DOCX)

S6 Table Identification of 71 proteins by shotgun proteomics in pacu fast-twitch muscle after 30 days of refeeding. Proteins were considered differently expressed between group C and E when fold change ≥1.5 or ≤0.6.
(DOCX)

S7 Table Western Blot measurements. The PVALB measurements were divided by β-actin measurements and were subsequently normalized to the mean of the control. Outlier values were eliminated from analysis.
(DOCX)

S8 Table miRWalk search for miRNAs targeting parvalbumin transcripts against human database. (XLSX)

S9 Table miRWalk search for miRNAs targeting parvalbumin transcripts against mouse database. (XLSX)

S10 Table miRWalk search for miRNAs targeting parvalbumin transcripts against rat database. (XLSX)

S11 Table Predicted miRNAs for each set of parvalbumin transcripts. (XLSX)

S1 Fig PVALB Western Blot of fast-twitch muscle of juvenile pacu after fasting and refeeding. β-actin was used as a loading control.
(DOCX)

S2 Fig Predicted interaction of the miRNA miR-23a-5p to the 3’ UTR region of Danio rerio pvalb2 transcript. The mRNA structure was produced with RNAstructure Web Server and the interaction between the miRNA and target was predicted with RNAHybrid.
(DOCX)

S3 Fig Predicted interaction of the miRNA miR-149-3p to the 3’ UTR region of Danio rerio pvalb2 transcript. The mRNA structure was produced with RNAstructure Web Server and the interaction between the miRNA and target was predicted with RNAHybrid.
(DOCX)

S1 Video 3D protein interaction network of differentially expressed proteins in the proteomic analysis after 30 days of fasting. The software MoNet was used to produce the video.
(MP4)

S2 Video 3D protein interaction network of differentially expressed proteins in the proteomic analysis after 30 days of refeeding. The software MoNet was used to produce the video.
(MP4)
