# Supplementary material for: Prolonged fasting followed by refeeding modifies proteome profile and parvalbumin expression in the fast-twitch muscle of pacu (Piaractus mesopotamicus)
Source: PLoS One. 2019 Dec 19;14(12):e0225864. doi: 10.1371/journal.pone.0225864 (PMC6922423; doi:10.1371/journal.pone.0225864)
Supplement: S1 Table — F–Forward sequence; R–Reverse sequence. (DOCX) [file pone.0225864.s001.docx]

**S1 Table –** Sequence of the primers used for quantitative real-time PCR (RT-qPCR) genes amplification in juveniles pacu (*Piaractus mesopotamicu*s) fast muscle. F – Forward sequence; R – Reverse sequence.

| Gene | Sequence (5’ – 3’) |
| --- | --- |
| *mafbx* | F – TCTTTGGTGCTCCCCTTGTG |
|  | R –TAAAACCGAGGACGGCTGG |
| *pvalb* | F – AGAAGAGGTTGTCGGGTTGC |
|  | R – GGATGGGGACGGGAAGATTG |
| *igf-1* | F-ATTTCAGCAAGCAACAGGT |
|  | R-CGCACAATAGATCTGAAGTCG |
| *rpl13* | F – ATCAACAGGAAAGTAGCCC |
|  | R –AGGATGAGTTTGGAGCGGTA |
