# Supplementary material for: Prolonged fasting followed by refeeding modifies proteome profile and parvalbumin expression in the fast-twitch muscle of pacu (Piaractus mesopotamicus)
Source: PLoS One. 2019 Dec 19;14(12):e0225864. doi: 10.1371/journal.pone.0225864 (PMC6922423; doi:10.1371/journal.pone.0225864)
Supplement: S2 Table — (DOCX) [file pone.0225864.s002.docx]

**S2 Table –** Descriptive data of weight (grams) of juvenile *Piaractus mesopotamicus* submitted to 30 days of fasting and 30 days of refeeding.

|  | **START OF EXPERIMENT** | **EXPERIMENTAL GROUP** | | **CONTROL GROUP** | |
| --- | --- | --- | --- | --- | --- |
|  |  | **Fasting** | **Refeeding** | **Fasting** | **Refeeding** |
| **Samples** | 90 g | 46.9 g | 81.6 g | 82.7 g | 103 g |
|  | 95.2 g | 63.6 g | 84.4 g | 106.6 g | 132.6 g |
|  | 107.9 g | 66.8 g | 87.4 g | 106.8 g | 143.6 g |
|  | 115.9 g | 75.3 g | 119.6 g | 117.3 g | 151.6 g |
|  | 117.7 g | 75.3 g | 123.3 g | 124.6 g | 160.6 g |
|  | 110.3 g | 77.7 g | 140.6 g | 128.1 g | 167.3 g |
|  | 113.3 g | 98.4 g | 144.2 g | 131.8 g | 171.5 g |
|  | 122.4 g | 110.5 g | 152.9 g | 139.6 g | 188.1 g |
|  |  | 112.8 g | 156.6 g | 257.4 g | 212.4 g |
| **Median** | 111.8 | 75.3 | 123.3 | 124.6 | 160.6 |
| **Mean** | 109.08 | 80.81 | 121.17 | 132.76 | 158.96 |
| **Standard deviation** | 11.18 | 22.15 | 30.07 | 49.71 | 31.72 |
| **RRI_weight_** | N/A | 25.92 | 49.95 | 21.70 | 19.73 |
