# Supplementary material for: Prolonged fasting followed by refeeding modifies proteome profile and parvalbumin expression in the fast-twitch muscle of pacu (Piaractus mesopotamicus)
Source: PLoS One. 2019 Dec 19;14(12):e0225864. doi: 10.1371/journal.pone.0225864 (PMC6922423; doi:10.1371/journal.pone.0225864)
Supplement: S3 Table — (DOCX) [file pone.0225864.s003.docx]

**S3 Table –** Descriptive data of length (centimetres) of juvenile *Piaractus mesopotamicus* submitted to 30 days of fasting and 30 days of refeeding.

|  | **START OF EXPERIMENT** | **EXPERIMENTAL GROUP** | | **CONTROL GROUP** | |
| --- | --- | --- | --- | --- | --- |
|  |  | **Fasting** | **Refeeding** | **Fasting** | **Refeeding** |
| **Samples** | 13.4 cm | 11.2 cm | 13 cm | 13.2 cm | 14.2 cm |
|  | 13.7 cm | 11.3 cm | 13.4 cm | 14.3 cm | 15.2 cm |
|  | 13.9 cm | 12.4 cm | 13.6 cm | 13.8 cm | 14.7 cm |
|  | 14.3 cm | 12.7 cm | 13.9 cm | 14.7 cm | 17.1 cm |
|  | 14.3 cm | 12.2 cm | 15 cm | 15 cm | 17 cm |
|  | 14.4 cm | 13 cm | 17.3 cm | 14.5 cm | 17.6 cm |
|  | 14.4 cm | 13.7 cm | 16.8 cm | 15.2 cm | 17.8 cm |
|  | 14.6 cm | 14.7 cm | 17.2 cm | 15.7 cm | 18.2 cm |
|  |  | 14.5 cm | 15.6 cm | 20.1 cm | 19.2 cm |
| **Median** | 14.3 | 12.7 | 15 | 14.7 | 17.1 |
| **Mean** | 14.125 | 12.85555556 | 15.08888889 | 15.16666667 | 16.77777778 |
| **Standard deviation** | 0.413175853 | 1.258084964 | 1.709857044 | 1.993740204 | 1.703509449 |
| **RRI_lenght_** | N/A | 8.987217306 | 17.37251513 | 7.374631268 | 10.62271062 |
