# Supplementary material for: Prolonged fasting followed by refeeding modifies proteome profile and parvalbumin expression in the fast-twitch muscle of pacu (Piaractus mesopotamicus)
Source: PLoS One. 2019 Dec 19;14(12):e0225864. doi: 10.1371/journal.pone.0225864 (PMC6922423; doi:10.1371/journal.pone.0225864)
Supplement: S4 Table — Gene expression was assessed with RT-qPCR. The fold-change was calculated by the comparative Ct method and is relative to the control rpl13. The same control was used for 6h and 24h of refeeding in order to minimize the number of slaughtered animals. (DOCX) [file pone.0225864.s004.docx]

**S4 Table –** Fold-change of *mafbx, igf-1* and *pvalb* in the fast-twitch muscle of *Piaractus mesopotamicus* after 30 days of fasting and 6h, 24h, 48h and 30 days of refeeding. Gene expression was assessed with RT-qPCR. The fold-change was calculated by the comparative Ct method and is relative to the control *rpl13*. The same control was used for 6h and 24h of refeeding in order to minimize the number of slaughtered animals.

|  |  | **CONTROL GROUP** | | | **EXPERIMENTAL GROUP** | | |
| --- | --- | --- | --- | --- | --- | --- | --- |
|  |  | **Mean** | **Standard deviation** | **Sample size** | **Mean** | **Standard deviation** | **Sample size** |
|  |  | ***mafbx*** | | | | | |
| **FASTING** | **30d** | 1.212 | 0.8143 | 9 | 3.546 | 1.62 | 8 |
| **REFEEDING** | **6h** | 1.212 | 0.8143 | 9 | 1.709 | 1.122 | 7 |
|  | **24h** | 1.212 | 0.8143 | 9 | 1.654 | 1.381 | 4 |
|  | **48h** | 1.203 | 0.7127 | 5 | 0.2522 | 0.125 | 5 |
|  | **30d** | 0.8527 | 0.228 | 4 | 0.7027 | 0.4376 | 7 |
|  |  | ***igf-1*** | | | | | |
| **FASTING** | **30d** | 1.054 | 0.3477 | 9 | 0.5269 | 0.2099 | 7 |
| **REFEEDING** | **6h** | 1.054 | 0.3477 | 9 | 0.4894 | 0.2821 | 9 |
|  | **24h** | 1.054 | 0.3477 | 9 | 0.4532 | 0.1629 | 5 |
|  | **48h** | 1.073 | 0.437 | 7 | 0.8834 | 0.4816 | 5 |
|  | **30d** | 1.054 | 0.3832 | 5 | 0.7949 | 0.472 | 8 |
|  |  | ***pvalb*** | | | | | |
| **FASTING** | **30d** | 0.8644 | 0.6986 | 7 | 0.4983 | 0.2685 | 9 |
| **REFEEDING** | **6h** | 0.8644 | 0.6986 | 7 | 0.1559 | 0.0689 | 9 |
|  | **24h** | 0.8644 | 0.6986 | 7 | 0.1561 | 0.1019 | 5 |
|  | **48h** | 1.287 | 0.907 | 7 | 0.7461 | 0.3695 | 4 |
|  | **30d** | 1.124 | 0.5953 | 5 | 0.5396 | 0.2196 | 7 |
