# Supplementary material for: Prolonged fasting followed by refeeding modifies proteome profile and parvalbumin expression in the fast-twitch muscle of pacu (Piaractus mesopotamicus)
Source: PLoS One. 2019 Dec 19;14(12):e0225864. doi: 10.1371/journal.pone.0225864 (PMC6922423; doi:10.1371/journal.pone.0225864)
Supplement: S5 Table — Proteins were considered differently expressed between group C and E when fold change ≥1.5 or ≤0.6. (DOCX) [file pone.0225864.s005.docx]

**S5 Table –** Identification of 99 proteins by shotgun proteomic in pacu fast-twitch muscle after 30 days of fasting. Proteins were considered differently expressed between group C and E when fold change ≥1.5 or ≤0.6.

| Protein name | Accession Number | Fold Change | Species |
| --- | --- | --- | --- |
| Beta-actin, partial | ADV57161.1 | 0.00 | *Ctenopharyngodon idella* |
| Cofilin-2 | XP_007240534.1 | 0.00 | *Astyanax mexicanus* |
| Myosin heavy chain, fast skeletal muscle-like | XP_010747852.1 | 0.40 | *Larimichthys crocea* |
| Myosin-1-like | XP_007241732.2 | 0.40 | *Astyanax mexicanus* |
| Parvalbumin, partial | ACP30426.1 | 0.50 | *Hypomesus transpacificus* |
| Myosin-6 | XP_003459542.1 | 0.60 | *Oreochromis niloticus* |
| Myosin-4-like | XP_014330160.1 | 0.60 | *Xiphophorus maculatus* |
| Myosin-7 isoform X1 | XP_008289363.1 | 0.60 | *Stegastes partitus* |
| Parvalbumin-like, partial | KPP74939.1 | 0.60 | *Scleropages formosus* |
| Fructose-bisphosphatealdolase A | XP_012693155.1 | 0.70 | *Clupea harengus* |
| Myosin-7B-like | XP_005805036.1 | 0.70 | *Xiphophorus maculatus* |
| Aldolase a, fructose-bisphosphate, a | AAH44379.1 | 0.80 | *Danio rerio* |
| Fast skeletal muscle troponin C | AAF78473.1 | 0.80 | *Danio rerio* |
| Myosin heavy chain, fast skeletal muscle-like | XP_005816990.1 | 0.80 | *Xiphophorus maculatus* |
| Myosin light chain 3 skeletal muscle-like, partial | KPP66310.1 | 0.80 | *Scleropages formosus* |
| Troponin T, fast skeletal muscle isoforms-like isoform X1 | XP_007228156.1 | 0.80 | *Astyanax mexicanus* |
| Beta actin, partial | ACR20031.1 | 0.90 | *Soleasenegalensis* |
| Beta-enolase | XP_007234192.1 | 0.90 | *Astyanax mexicanus* |
| Beta-enolase-like | XP_012690312.1 | 0.90 | *Clupea harengus* |
| Creatine kinase M2-CK | AAC96093.1 | 0.90 | *Cyprinus carpio* |
| Enolase isoform X1 | XP_010902625.1 | 0.90 | *Esox lucius* |
| Glyceraldehyde phosphatede hydrogenase | AAK49985.1 | 0.90 | *Oncorhynchu smykiss* |
| Hypothetical protein cypcar_00032582, partial | KTF93862.1 | 0.90 | *Cyprinus carpio* |
| Myosin light chain3 | BAA95139.1 | 0.90 | *Sardinops melanostictus* |
| Parvalbumin, partial | AAO25757.1 | 0.90 | *Ictalurus punctatus* |
| Creatine kinase M-type | XP_013871967.1 | 1.00 | *Austrofundulus limnaeus* |
| Creatine kinase M-type-like | KPP72043.1 | 1.00 | *Scleropages formosus* |
| Creatine kinase M-type-like | XP_008302105.1 | 1.00 | *Stegastes partitus* |
| Golgin subfamily A member 4-like | XP_012682153.1 | 1.00 | *Clupea harengus* |
| Hypothetical protein Z043_125198, partial | KPP57110.1 | 1.00 | *Scleropages formosus* |
| Hypothetica lprotein cypcar_00004643 | KTG44280.1 | 1.00 | *Cyprinus carpio* |
| Myosin heavy chain | CAC27778.1 | 1.00 | *Notothenia coriiceps* |
| Myosin heavy chain | BAG16354.1 | 1.00 | *Coryphaenoides cinereus* |
| Myosin heavy chain, fast skeletal muscle | XP_005745390.1 | 1.00 | *Pundamilia nyererei* |
| Myosin heavy chain, partial | ACO51249.1 | 1.00 | *Paralichthys olivaceus* |
| Myosin light chain1 | BAB69806.1 | 1.00 | *Scomber japonicus* |
| Myosin light chain 3 skeletal muscle isoform | ADO28239.1 | 1.00 | *Ictaluru sfurcatus* |
| Myosin light chain3, skeletal muscle isoform | XP_003453231.1 | 1.00 | *Oreochromis niloticus* |
| Myosin light chain3, skeletal muscle isoform | XP_013875601.1 | 1.00 | *Austrofundulus limnaeus* |
| Myosin light polypeptide | AHI42526.1 | 1.00 | *Campylomormyrus compressirostris* |
| Myosin-3 | XP_015211512.1 | 1.00 | *Lepisosteusoculatus* |
| Skeletal muscle myosin heavy chain | ACA33869.1 | 1.00 | *Ctenopharyngodon idella* |
| Triosephosphate isomerase, partial | ACO57606.1 | 1.00 | *Gillichthys mirabilis* |
| Unnamed protein product | CDQ72325.1 | 1.00 | *Oncorhynchus mykiss* |
| Adenylate kinase isoenzyme1 | XP_007239674.1 | 1.10 | *Astyanax mexicanus* |
| Alpha actin | AAC59894.1 | 1.10 | *Takifuguru bripes* |
| Alpha actin, partial | ABN58888.1 | 1.10 | *Oreochromis niloticus* |
| Hypothetical protein cypcar_00046947 | KTF81810.1 | 1.10 | *Cyprinus carpio* |
| Myosin heavy chain | CAC59753.1 | 1.10 | *Paracirrhites forsteri* |
| Myosin heavy chain | ABC42922.2 | 1.10 | *Dicentrarchus labrax* |
| Myosin heavy chain | BAB12571.1 | 1.10 | *Pennahia argentata* |
| Myosin heavy chain embryonic type 1 | BAH70477.1 | 1.10 | *Oryzias latipes* |
| Myosin heavy chain, fast skeletal muscle | XP_011612179.1 | 1.10 | *Takifuguru bripes* |
| Myosin heavy chain, fast skeletal muscle | KKF26630.1 | 1.10 | *Larimichthys crocea* |
| Myosin heavy chain, fast skeletal muscle isoform X1 | XP_005173989.1 | 1.10 | *Danio rerio* |
| Myosin heavy chain, fast skeletal muscle-like | XP_015243945.1 | 1.10 | *Cyprino donvariegatus* |
| Myosin heavy chain, fast skeletal muscle-like | XP_006635175.2 | 1.10 | *Lepisosteus oculatus* |
| Myosin heavy chain, fast skeletal muscle-like | XP_015457104.1 | 1.10 | *Astyanax mexicanus* |
| Myosin light chain1 | BAA95129.1 | 1.10 | *Pennahia argentata* |
| Myosin light chain 1, skeletal muscle isoforms | P82159.1 | 1.10 |  |
| Myosin light chain 1/3, skeletal muscle isoform | XP_013866991.1 | 1.10 | *Austrofundulus limnaeus* |
| Myosin light chain 1/3, skeletal muscle isoform | XP_007557170.1 | 1.10 | *Poecilia formosa* |
| Myosin light chain2 | BAA95134.1 | 1.10 | *Cheilopogonagoo* |
| Myosin light chain-2 | AGU16240.1 | 1.10 | *Tanakiakoreensis* |
| Myosin regulatory light chain2, skeletal muscle isoforms | XP_007235411.1 | 1.10 | *Astyanaxmexicanus* |
| Triosephosphateisomerase b | ADO27908.1 | 1.10 | *Ictalurus furcatus* |
| Tropomyosin alpha-1 chain isoform X1 | XP_007239611.1 | 1.10 | *Astyanax mexicanus* |
| Tropomyosin alpha-1 chain isoform X3 | XP_007246561.1 | 1.10 | *Astyanax mexicanus* |
| Tropomyosin alpha-1 chain-like isoform X2 | XP_014004080.1 | 1.10 | *Salmo salar* |
| Tropomyosin alpha-4 chain-like, partial | KPP76376.1 | 1.10 | *Scleropages formosus* |
| Actin, aortic smooth muscle-like | XP_006642710.1 | 1.20 | *Lepisosteus oculatus* |
| Creatine kinase-1 | ACH70913.1 | 1.20 | *Salmo salar* |
| Myosin heavy chain, fast skeletal muscle-like | XP_012728166.1 | 1.20 | *Fundulus heteroclitus* |
| Tropomyosin | BAJ11924.1 | 1.20 | *Thunnus thynnus* |
| Tropomyosin alpha-4 chain | XP_011485407.1 | 1.20 | *Oryzias latipes* |
| Tropomyosin alpha-4 chain, partial | AER42688.1 | 1.20 | *Epinephe luscoioides* |
| Actin, cytoplasmic 2-like | KPP63191.1 | 1.30 | *Scleropages formosus* |
| Alpha-enolase | ACI33096.1 | 1.30 | *Salmo salar* |
| Beta actin, partial | ABR13252.1 | 1.30 | *Atherina boyeri* |
| Glyceraldehyde 3-phosphate dehydrogenase | BAF43305.1 | 1.30 | *Misgurnusan guillicaudatus* |
| Unnamed protein product, partial | CAG12586.1 | 1.30 | *Tetraodonni groviridis* |
| Beta-actin, partial | AJE28003.1 | 1.40 | *Alburno idessp. 4 JM-2015* |
| Glyceraldehyde-3-phosphate dehydrogenase, partial | AAG13319.1 | 1.40 | *Gillichthy smirabilis* |
| Glycogen phosphorylase, muscle form-like | XP_012686253.1 | 1.40 | *Clupeaha rengus* |
| Hypothetical protein cypcar_00046186 | KTF75169.1 | 1.40 | *Cyprinus carpio* |
| Beta-actin, partial | AJE28000.1 | 1.50 | *Alburnoides fasciatus* |
| Glyceraldehyde-3-phosphate dehydrogenase | XP_007249860.1 | 1.50 | *Astyanax mexicanus* |
| Sarcoplasmic/endoplasmic reticulum calcium atpase 1ª | AAB08097.1 | 1.50 | *Makaira nigricans* |
| Phosphorylase, glycogen (muscle) A | AAH95379.1 | 1.70 | *Danio rerio* |
| Phosphoglyceratemutase2 (muscle) | AAH53127.1 | 1.80 | *Danio rerio* |
| Tropomyosin alpha-1 chain isoform X4 | XP_014031659.1 | 1.80 | *Salmo salar* |
| Actinin alpha 3b | AAH65595.1 | 2.00 | *Danio rerio* |
| Alpha-actinin-1 | ACN10704.1 | 2.00 | *Salmo salar* |
| Creatine kinase muscle b | AHI42553.1 | 2.50 | *Campylomormyrus compressirostris* |
| Unnamed protein product | CAG06908.1 | 2.70 | *Tetraodonni groviridis* |
| Myosin heavy chain, fast skeletal muscle-like | XP_003442546.1 | 2.80 | *Oreochromis niloticus* |
| Alpha globin, partial | AAM93257.1 | 3.50 | *Ctenopharyngo donidella* |
| Beta-actin, partial | AAG43381.1 | 5.00 | *Coryphaenoi desarmatus* |
| Myosin-7-like | XP_015196754.1 | 5.00 | *Lepisosteus oculatus* |
