# Supplementary material for: Prolonged fasting followed by refeeding modifies proteome profile and parvalbumin expression in the fast-twitch muscle of pacu (Piaractus mesopotamicus)
Source: PLoS One. 2019 Dec 19;14(12):e0225864. doi: 10.1371/journal.pone.0225864 (PMC6922423; doi:10.1371/journal.pone.0225864)
Supplement: S6 Table — Proteins were considered differently expressed between group C and E when fold change ≥1.5 or ≤0.6. (DOCX) [file pone.0225864.s006.docx]

**S6 Table -** Identification of 71 proteins by shotgun proteomics in pacu fast-twitch muscle after 30 days of refeeding. Proteins were considered differently expressed between group C and E when fold change ≥1.5 or ≤0.6.

| Protein name | Accession  Number | Fold Change | Species |
| --- | --- | --- | --- |
| Myosin light chain 1, skeletal muscle isoform | XP_005800770.1 | 0.00 | *Xiphophorus maculatus* |
| Myosin heavy chain, fast skeletal muscle-like | XP_003442546.1 | 0.00 | *Oreochromis niloticus* |
| Myosin heavy chain | AAG13334.1 | 0.00 | *Gillichthys mirabilis* |
| Tropomyosin alpha-4 chain-like | XP_013887991.1 | 0.20 | *Austrofundulus limnaeus* |
| Cratine kinase muscle b | AHI42553.1 | 0.20 | *Campylomormyrus compressirostris* |
| Alpha tropomyosin | AAG13297.1 | 0.30 | *Gillichthysmirabilis* |
| Myosin-7 isoform X1 | XP_008289363.1 | 0.40 | *Stegastes partitus* |
| Parvalbumin alpha | P09227.2 | 0.50 | *Cyprinus carpio* |
| MAX gene-associatedprotein | XP_015824795.1 | 0.50 | *Nothobranchius furzeri* |
| Creatine kinase-1 | ACH70913.1 | 0.60 | *Salmo salar* |
| Titin-like | XP_015248196.1 | 0.60 | *Cyprino donvariegatus* |
| Tropomyosin alpha-1 chain isoform X4 | XP_014031659.1 | 0.70 | *Salmo salar* |
| Hypothetical protein cypcar_00002572 | KTG05890.1 | 0.70 | *Cyprinus carpio* |
| Tropomyosin alpha-1 chain-like isoform X2 | XP_014004080.1 | 0.80 | *Salmo salar* |
| Tropomyosin | BAJ11924.1 | 0.80 | *Thunnus thynnus* |
| Tropomyosin alpha-4 chain | AER42688.1 | 0.80 | *Epinephelus coioides* |
| Tropomyosin alpha-4 chain-like | KPP76376.1 | 0.80 | *Scleropages formosus* |
| Tropomyosin alpha-4 chain-like isoform X2 | XP_010883431.1 | 0.80 | *Esox lucius* |
| Zgc:103639 | AAH81501.1 | 0.80 | *Danio rerio* |
| Myosin light chain 3 skeletal muscle isoform | ADO28239.1 | 0.90 | *Ictalurus furcatus* |
| Myosin light chain 1/3, skeletal muscle isoform | XP_013866991.1 | 0.90 | *Austrofundulus limnaeus* |
| Tropomyosin alpha-1 chain isoform X1 | XP_007239611.1 | 0.90 | *Astyanax mexicanus* |
| Tropomyosin alpha-1 chain isoform X3 | XP_007246561.1 | 0.90 | *Astyanax mexicanus* |
| Myosin heavy chain, fast skeletal muscle-like | XP_015814305.1 | 0.90 | *Nothobranchius furzeri* |
| Myosin heavy chain, fast skeletal muscle | XP_005745390.1 | 0.90 | *Pundamilian yererei* |
| Fast skeletal myosin heavy chain isoform mmyh-1 | BAF34701.1 | 0.90 | *Oryzias latipes* |
| Hypothetical protein Z043_125198, partial | KPP57110.1 | 0.90 | *Scleropages formosus* |
| Myosin heavy chain | CAC27778.1 | 0.90 | *Notothenia coriiceps* |
| Creatine kinase M-type-like | KPP72043.1 | 0.90 | *Scleropages formosus* |
| Adenylate kinase isoenzyme1 | XP_007239674.1 | 0.90 | *Astyanax mexicanus* |
| Triosephosphate isomerase | ACO57606.1 | 0.90 | *Gillichthys mirabilis* |
| Parvalbumin beta | P02618.1 | 0.90 | *Cyprinus carpio* |
| Triosephosphate isomerase b | ADO27908.1 | 0.90 | *Ictalurus furcatus* |
| Myosin light chain 3, skeletal muscle isoform | XP_003453231.1 | 1.00 | *Oreochromis niloticus* |
| Myosin light chain 1, skeletal muscle isoform | XP_008327037.1 | 1.00 | *Cynoglossus semilaevis* |
| Myosin light chain 1, skeletal muscle isoform | XP_010742745.1 | 1.00 | *Larimichthys crocea* |
| Myosin light chain 1, skeletal muscle isoform | XP_003445346.1 | 1.00 | *Oreochromis niloticus* |
| Myosin light chain1, skeletal muscle isoform | ACQ59062.1 | 1.00 | *Anoplopoma fimbria* |
| Myosin heavy chain, fast skeletal muscle-like | XP_015243945.1 | 1.00 | *Cyprinod onvariegatus* |
| Myosin heavy chain, fast skeletal muscle | XP_012728167.1 | 1.00 | *Fundulus heteroclitus* |
| Myosin heavy chain a | AGH09216.1 | 1.00 | *Culteral burnus* |
| Myosin heavy chain, fast skeletal muscle-like | XP_005816990.1 | 1.00 | *Xiphophorus maculatus* |
| Myosin heavy chain | BAG16353.1 | 1.00 | *Coryphaenoides macrolepis* |
| Myosin heavy chain, fast skeletal muscle-like | XP_012728166.1 | 1.00 | *Fundulus heteroclitus* |
| Fast skeletal myosin heavy chain isoform mmyh-7 | BAF34704.1 | 1.00 | *Oryzias latipes* |
| Myosin heavy chain-1 | BAL27685.1 | 1.00 | *Thunnus orientalis* |
| Myosin heavy chain, fast skeletal muscle | XP_003976959.1 | 1.00 | *Takifuguru bripes* |
| Myosin heavy chain embryonic type 1 | BAH70477.1 | 1.00 | *Oryzias latipes* |
| Myosin heavy chain | BAF49657.1 | 1.00 | *Sauridaundos quamis* |
| Myosin regulatory light chain 2, skeletal muscle isoform | XP_015804253.1 | 1.00 | *Nothobranchius furzeri* |
| Myosin regulatory light chain 2, skeletal muscle isoform | XP_007235411.1 | 1.00 | *Astyanax mexicanus* |
| Creatine kinase M2-CK | AAC96093.1 | 1.00 | *Cyprinus carpio* |
| Creatine kinase M-type | XP_013871967.1 | 1.00 | *Austrofundulus limnaeus* |
| Troponin I, fast skeletal muscle-like | XP_007228170.1 | 1.00 | *Astyanax mexicanus* |
| Myosin light chain 3 skeletal muscle-like | KPP66310.1 | 1.10 | *Scleropagesf ormosus* |
| Myosin light chain1 | BAB69806.1 | 1.10 | *Scomberj aponicus* |
| Myosin light chain 3, skeletal muscle isoform | XP_013875601.1 | 1.10 | *Austrofundulus limnaeus* |
| Myosin heavy chain | AAS19755.1 | 1.10 | *Gasterosteus aculeatus* |
| Troponin T, fast skeletal muscle isoforms-like isoform X1 | XP_007228156.1 | 1.10 | *Astyanax mexicanus* |
| Myosin light chain 1, skeletal muscle isoform | XP_007234897.1 | 1.20 | *Astyanax mexicanus* |
| Unnamed protein product, partial | CAG12586.1 | 1.20 | *Tetraodonni groviridis* |
| Myosin light polypeptide 6-like | XP_007248797.1 | 1.20 | *Astyanax mexicanus* |
| Myosin light chain3 | BAA95139.1 | 1.30 | *Sardinops melanostictus* |
| Myosin heavy chain, fast skeletal muscle-like | XP_015257849.1 | 1.30 | *Cyprino donvariegatus* |
| Beta-enolase-like | XP_012690312.1 | 1.40 | *Clupea harengus* |
| Beta-actin | AJE27970.1 | 1.50 | *Alburnoides bipunctatus* |
| Hypothetical protein cypcar_00032582 | KTF93862.1 | 1.50 | *Cyprinus carpio* |
| Alpha-enolase | ACI33096.1 | 1.50 | *Salmo salar* |
| Myosin heavy chain | ACO51249.1 | 1.50 | *Paralichthys olivaceus* |
| Enolase isoform X1 | XP_010902625.1 | 2.10 | *Esox lucius* |
| Fructose-bisphosphate aldolase A | XP_007248760.1 | 2.60 | *Astyanax mexicanus* |
