# Supplementary material for: Prolonged fasting followed by refeeding modifies proteome profile and parvalbumin expression in the fast-twitch muscle of pacu (Piaractus mesopotamicus)
Source: PLoS One. 2019 Dec 19;14(12):e0225864. doi: 10.1371/journal.pone.0225864 (PMC6922423; doi:10.1371/journal.pone.0225864)
Supplement: S7 Table — The PVALB measurements were divided by β-actin measurements and were subsequently normalized to the mean of the control. Outlier values were eliminated from analysis. (DOCX) [file pone.0225864.s007.docx]

**S7 Table –**Western Blot measurements. The PVALB measurements were divided by β-actin measurements and were subsequently normalized to the mean of the control. Outlier values were eliminated from analysis.

|  | **CONTROL GROUP** | | | | | | **EXPERIMENTAL GROUP** | | | | | |
| --- | --- | --- | --- | --- | --- | --- | --- | --- | --- | --- | --- | --- |
| **fasting** | 0.9300 | 1.086 | 1.0250 | 0.9580 |  |  | 0.8040 | 0.8880 | 0.6280 | 0.6230 | 0.9760 |  |
| **Refeeding** | 0.8853 | 0.9313 | 1.0183 | 0.7460 | 1.1133 | 1.3055 | 0.8851 | 0.6463 | 0.5476 | 0.7340 | 0.6828 | 0.6436 |
