# Supplementary material for: Prolonged fasting followed by refeeding modifies proteome profile and parvalbumin expression in the fast-twitch muscle of pacu (Piaractus mesopotamicus)
Source: PLoS One. 2019 Dec 19;14(12):e0225864. doi: 10.1371/journal.pone.0225864 (PMC6922423; doi:10.1371/journal.pone.0225864)
Supplement: S1 Fig — β-actin was used as a loading control. (DOCX) [file pone.0225864.s012.docx]

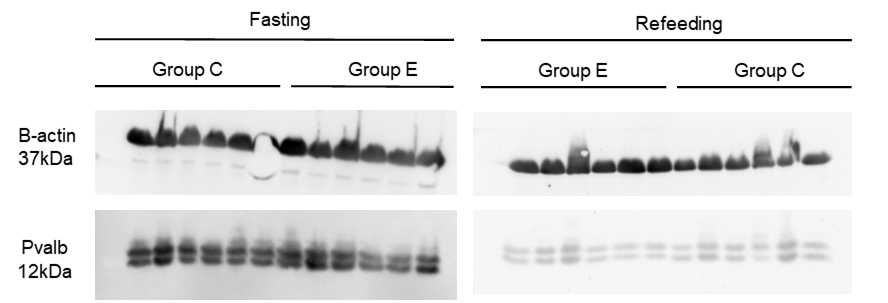


**S1 Figure –** PVALB Western Blot of fast-twitch muscle of juvenile pacu after fasting and refeeding. β-actin was used as a loading control.
