# Supplementary material for: Prolonged fasting followed by refeeding modifies proteome profile and parvalbumin expression in the fast-twitch muscle of pacu (Piaractus mesopotamicus)
Source: PLoS One. 2019 Dec 19;14(12):e0225864. doi: 10.1371/journal.pone.0225864 (PMC6922423; doi:10.1371/journal.pone.0225864)
Supplement: S2 Fig — The mRNA structure was produced with RNAstructure Web Server and the interaction between the miRNA and target was predicted with RNAHybrid. (DOCX) [file pone.0225864.s013.docx]

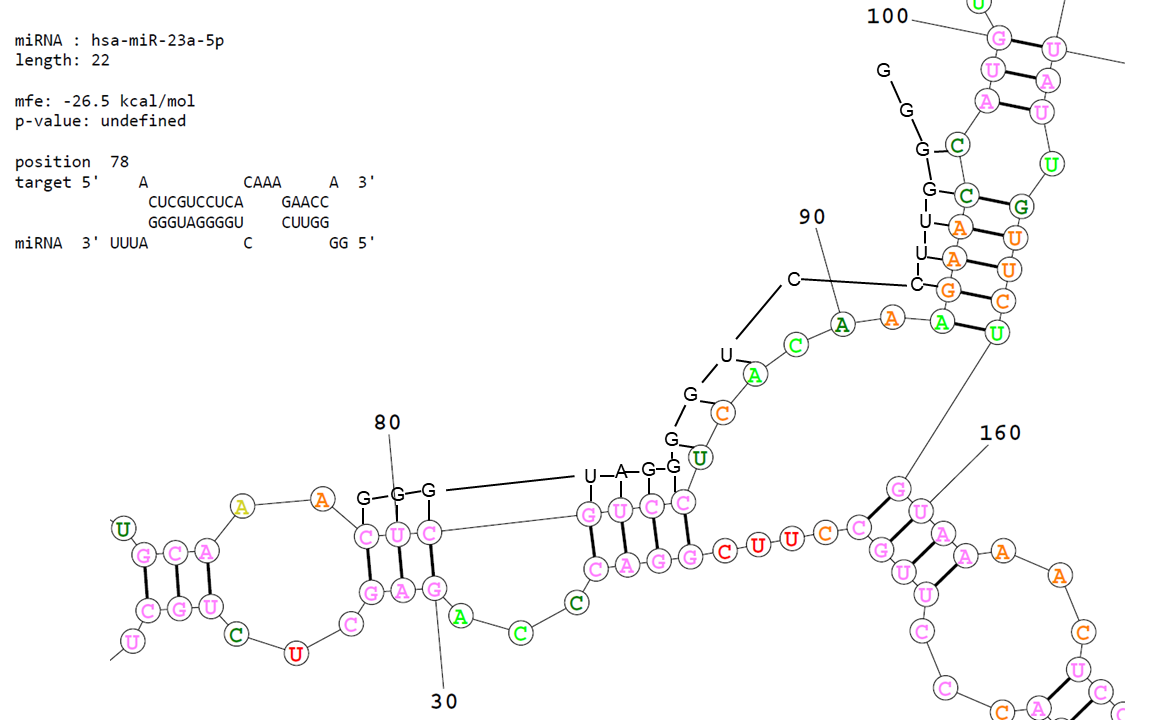


**S2 Figure –** Predicted interaction of the miRNA miR-23a-5p to the 3’ UTR region of Danio rerio pvalb2 transcript. The mRNA structure was produced with RNAstructure Web Server and the interaction between the miRNA and target was predicted with RNAHybrid.
